# Masquelet technique in military practice: specificities and future directions for combat-related bone defect reconstruction

**DOI:** 10.1186/s40779-022-00411-1

**Published:** 2022-09-02

**Authors:** Laurent Mathieu, Romain Mourtialon, Marjorie Durand, Arnaud de Rousiers, Nicolas de l’Escalopier, Jean-Marc Collombet

**Affiliations:** 1 Department of Orthopedic, Trauma and Reconstructive Surgery, Percy Military Hospital, 101 avenue Henri Barbusse, 92140 Clamart, France; 2 Department of Surgery, Ecole du Val-de-Grâce, French Military Health Service Academy, 1 place Alphonse Laveran, 75005 Paris, France; 3 Military Biomedical Research Institute (IRBA), 1 place Général Valérie André, 91220 Brétigny-sur-Orge, France

**Keywords:** Bone defect, Induced membrane technique, Gunshot wound, Low resources, Masquelet technique, Military, War surgery

## Abstract

Because of its simplicity, reliability, and replicability, the Masquelet induced membrane technique (IMT) has become one of the preferred methods for critical bone defect reconstruction in extremities. Although it is now used worldwide, few studies have been published about IMT in military practice. Bone reconstruction is particularly challenging in this context of care due to extensive soft-tissue injury, early wound infection, and even delayed management in austere conditions. Based on our clinical expertise, recent research, and a literature analysis, this narrative review provides an overview of the IMT application to combat-related bone defects. It presents technical specificities and future developments aiming to optimize IMT outcomes, including for the management of massive multi-tissue defects or bone reconstruction performed in the field with limited resources.

## Background

The reconstruction of segmental bone defects remains a challenge for military orthopedic surgeons, especially in cases of missile or blast injuries characterized by a high proportion of multi-tissue and infected defects [1–5]. Treatment modalities include techniques such as autologous bone graft (ABG), bone transport, vascularized bone transfer, and the two-stage Masquelet induced membrane (IM) technique (IMT; Table 1) [6, 7]. Because of its simplicity, reliability, and efficiency, even for large defects, the latter has spread globally in the last decade [8, 9]. The IMT presents strong advantages because it requires no sophisticated equipment or microsurgical skills to perform and has a healing time almost independent of the defect length [3]. This simplicity makes it particularly adapted to bone reconstruction in military practice, especially in current high-intensity warfare where patients cannot be evacuated from the combat zone or present with multiple associated injuries [2, 3, 5, 10].

**Table 1:** Strategies for bone defect reconstruction according to the French Society of Orthopedics (*Société Française de Chirurgie Orthopédique et Traumatologique*—SOFCOT) [6]

	Type 1 defect (length ≤ 2 cm)	Type 2 defect (2 cm < length ≤ 5 cm)	Type 3 defect (5 cm < length ≤ 10 cm)	Type 4 defect (length > 10 cm)
Humerus	Shortening or ABG	Shortening + ABG or IMT or bone transport	IMT or VBT	IMT or VBT
Forearm	ABG	IMT	IMT or VBT	OBF + IMT or OBF + VBT
Femur	ABG	IMT or ABG	IMT or VBT or bone transport	
Tibia	ABG including ITF grafting	IMT or ITF grafting or VBT or bone transport	IMT or VBT or bone transport or fibula tibialisation	

The SOFCOT classification is based on the defect length but does not include limb length discrepancy. *ABG* autologous bone graft, *IMT* induced membrane technique, *ITF* inter-tibiofibular, *OBF* one bone forearm procedure, *VBT* vascularized (fibular) bone transfer

**Fig. 1:**
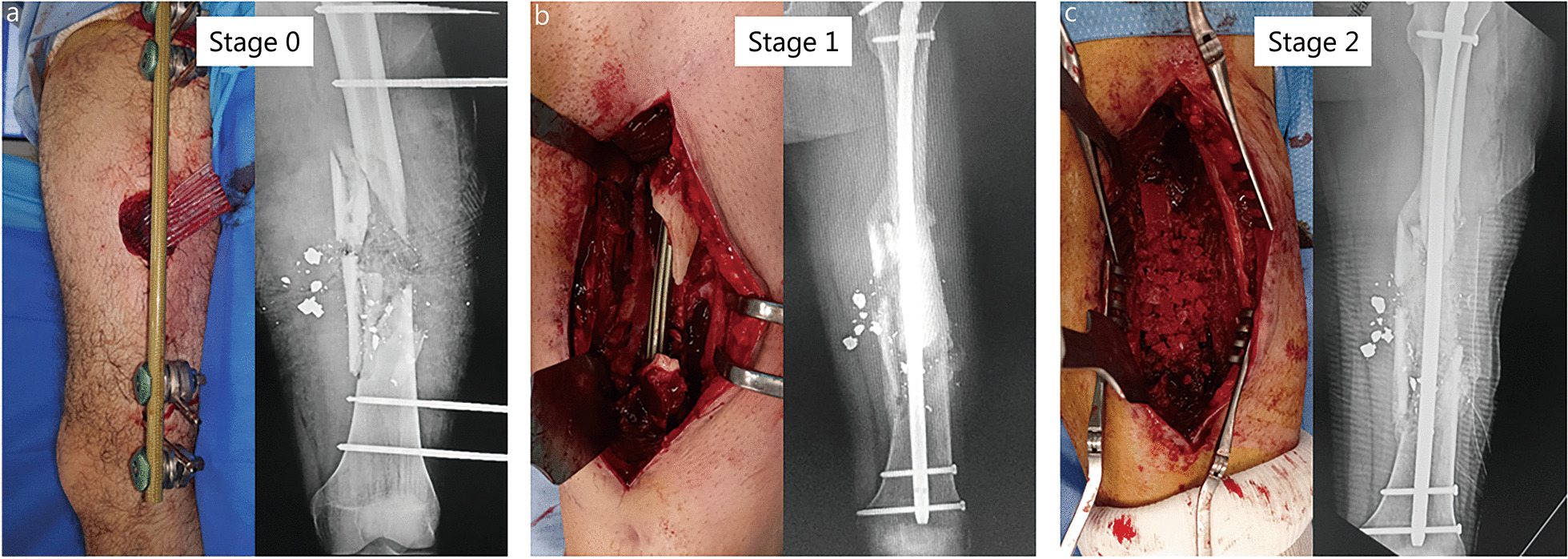
Management of an 11-cm femur defect (SOFCOT type 4) due to gunshot. **a** Wound debridement, temporary external fixation, and delayed primary closure according to DCO principles. **b** One-step conversion to intramedullary nailing at stage 1. **c** Cancellous bone grafting at stage 2. Bone union was achieved after 4 months with an excellent functional result (no pain or knee stiffness). DCO damage control orthopedics. SOFCOT *Société Française de Chirurgie Orthopédique et Traumatologique*

IMT is a two-stage procedure, using a cement spacer in the first stage and an ABG in the second stage. The spacer has a mechanical action, obviating fibrous tissue invasion of the recipient site, and a biological action that induces the surrounding membrane due to a foreign body reaction. The membrane then acts as a biological chamber that revascularizes the bone graft and prevents resorption [8, 9]. We believe that these two stages are beneficial when dealing with the large, multi-tissue, and infected defects encountered in wartime [2, 10]. Reconstruction of combat-related extremity injuries occurs in the aftermath of a primary damage control orthopedics (DCO) procedure and requires a sequential surgical approach to achieve infection control, soft-tissue coverage, and bone union (Fig. 1). In this context of care, the IMT matches perfectly with such consecutive reconstructive procedures [5, 11].

To date, only a few authors have reported on the Masquelet technique for reconstruction of combat-related bone defects [3, 12–14]. In 2019, we published the first clinical series about tibia reconstruction by IMT in soldiers [3]. Since then, we have repeatedly published on IMT use in the various settings of our military practice. Like civilian authors, we have found that this technique achieves satisfying outcomes in bone union and complication rates [3, 12, 15–22]. We have also demonstrated that IMT is suited for bone defects of ballistic origin and can be applied in the austere environment of forward surgical units [12, 17, 22]. However, although simple, a successful IMT procedure is not easy to complete, especially when facing infected defects with limited resources [3, 18, 22]. Therefore, this review aims to present Masquelet technique tips and tricks for the reconstruction of combat-related bone defects in modern warfare.

## Masquelet technique basics

### How does it work?

#### Foreign body encapsulation membrane

The IM is an encapsulation membrane resulting from a foreign body reaction caused by the implantation of a polymethylmethacrylate (PMMA) cement spacer into the bone defect. This foreign body reaction, composed of macrophages and foreign body giant cells, is the end-stage response of the inflammatory and wound healing responses following implantation of any medical device or biomaterial [23]. The IM is a vascularized collagen-based matrix entrapping immune cells (macrophages, lymphocytes), as well as bone-remodeling osteoclasts and osteoprogenitor cells (mesenchymal stem cells) [24, 25].

#### IM biological properties

From a biological perspective, the IM is a tissue regeneration chamber promoting bone graft integration and bone formation in three ways. First, the membrane acts as a physical barrier between the graft and the surrounding soft tissues, preventing macrophage invasion and subsequent graft resorption. Next, the membrane improves both angiogenesis and osteogenesis by secreting key growth factors including vascular endothelial growth factor (VEGF), transforming growth factor-β1 (TGF-β1), and bone morphogenic protein-2 (BMP-2) [26]. Briefly, VEGF-A stimulates the formation of vascular buds on the inner surface of the membrane, which penetrate bone graft crevices and permit effective graft revascularization [27]. BMP-2 and TGF-β1 stimulate bone formation by promoting the osteogenic differentiation of bone marrow stem cells [28].

### Prerequisites for use

Masquelet [9] and Giannoudis et al. [7] have already detailed technical specificities for a successful IMT application. However, it should be stressed that three conditions are imperative to prevent IMT failure: prior infection eradication, appropriate soft-tissue coverage during stage 1, and stable bone fixation during stage 2.

#### Prior infection eradication

Infection is the single most frequent cause of IMT failure [21]. Since they have no individual vascularization, bone grafts are directly affected by the presence of germs within the membrane, leading to subsequent graft resorption or late septic recurrence. In addition, infection alters IM osteogenic properties by decreasing local expression of growth factors, decreasing osteoblast and stem cell presence, and increasing bone resorption factors and proinflammatory cytokines [29, 30]. Thus, IMT must only be deployed once any infection is definitively eradicated or at least brought under control. In cases of infected bone defect, a prior IMT stage 0 is then crucial before cement spacer implantation [4, 31]. Stage 0 includes multiple consecutive procedures: removal of eventual internal fixation material, radical debridement of infected tissue, copious irrigation, and temporary stabilization by external fixation. Deep samples should be taken from the bone defect area and the medullary canal for microbiological analysis [4].

#### Appropriate soft-tissue coverage at stage 1

**Fig. 2:**
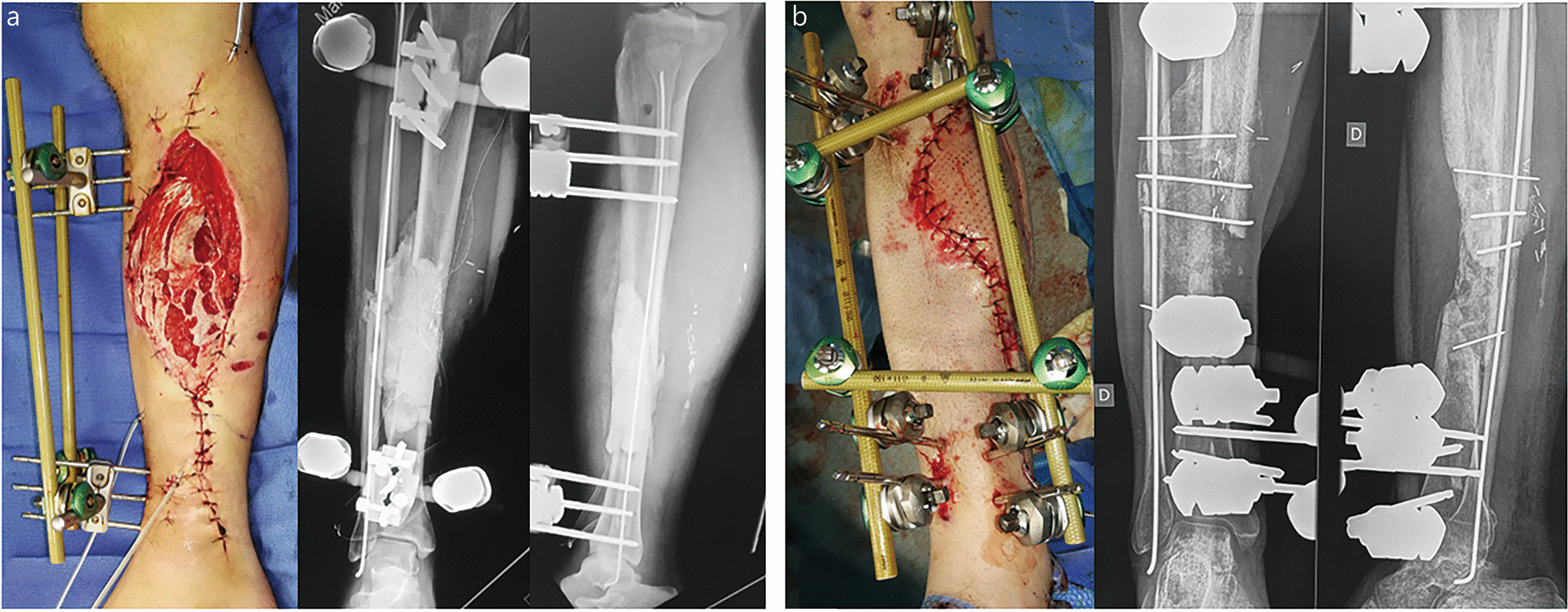
Reconstruction of a 10 cm infected diaphyseal tibia defect (SOFCOT type 4) using external fixation. **a** Stage 1: unilateral tibial frame combined with internal fixation of the fibula together with implantation of an inter-tibiofibular spacer and soleus flap transfer. **b** Stage 2: conversion to a multiplanar tibial frame with cancellous bone grafting and double inter-tibiofibular grafting. Bone union was achieved at month-10 after a progressive external fixator dynamization. No septic recurrence was observed at the last follow-up. SOFCOT *Société Française de Chirurgie Orthopédique et Traumatologique*

**Fig. 3:**
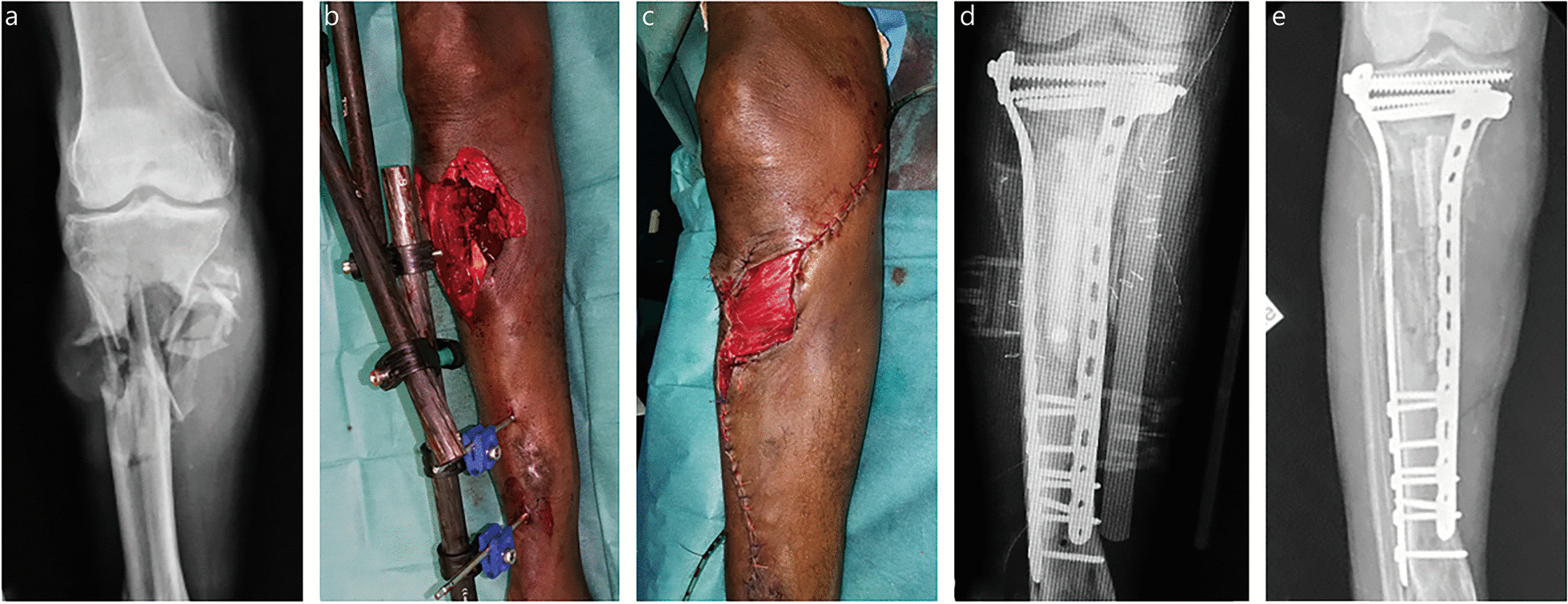
Reconstruction of a proximal tibia defect (SOFCOT type 3) in a local patient managed by a forward surgical team. **a** Radiological view of the gunshot wound at the admission. **b** Primary management according to DCO principles. **c**, **d** Conversion to definitive plating, implantation of a 9 cm length spacer, and coverage by a medial gastrocnemius muscle flap at stage 1. **e** Radiological view after autografting using cancellous bone grafts and a non-vascularized fibula strut. Bone union was achieved after 6 months but the patient suffered from knee stiffness due to the absence of physical therapy. DCO damage control orthopedics, SOFCOT *Société Française de Chirurgie Orthopédique et Traumatologique*

Cement spacer implantation requires not only the absence of infection but also stable soft-tissue coverage. Any secondary skin necrosis or wound dehiscence inevitably leads to cement spacer contamination, with subsequent infection and IMT failure. Thus, management of combat-related bone defects using the Masquelet technique often involves soft-tissue reconstruction using flap transfer during stage 1. The choice between micro-vascularized free flaps and pedicled (local or distant) flaps depends on the context of care, surgical resources, and associated injuries. Free flaps are more suited to stable patients managed in the ideal conditions of medical treatment facilities located outside the combat zone [1, 2]. Conversely, pedicled flaps are needed for early soft-tissue coverage in polytraumatized patients and in the austere conditions of forward surgical units where plastic surgeons are few or absent (Figs. 2 and 3) [4, 10, 32].

#### Stable bone fixation at stage 2

**Fig. 4:**
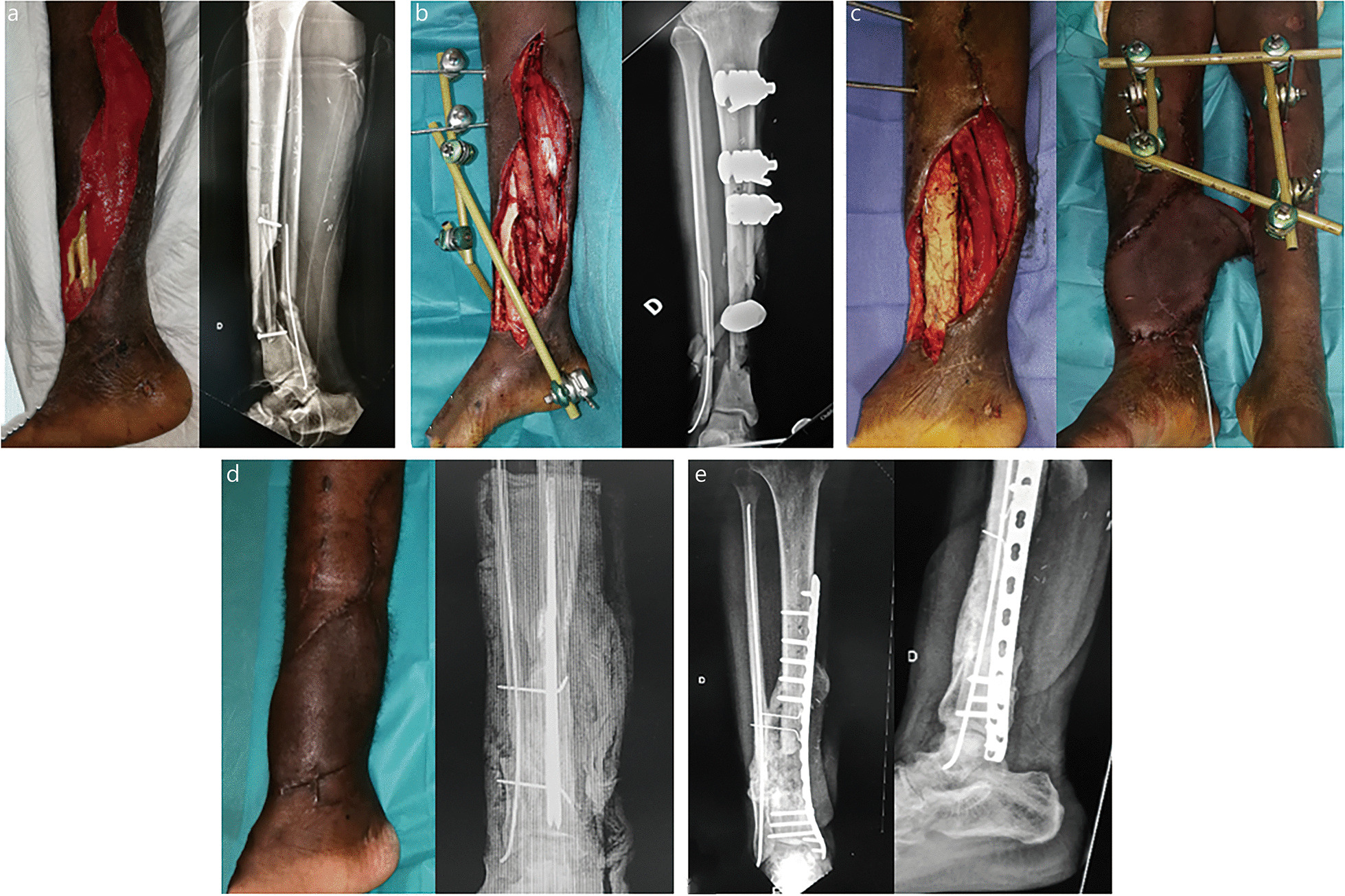
Reconstruction of an infected distal tibia defect (SOFCOT type 4) using sequential internal fixation. **a** Clinical and radiological view after a failed reconstruction using a composite vascularized fibula transfer. **b** Debridement and temporary external fixation before Masquelet technique application (stage 0). **c** Implantation of an 11 cm-long reinforced spacer and revision flap coverage using a pedicled cross-leg flap at stage 1. **d** Clinical and radiological view after division of the cross-leg flap. **e** Bone union after plating and bone grafting. At the last follow-up of 2 years, the functional outcome was excellent without septic recurrence. SOFCOT *Société Française de Chirurgie Orthopédique et Traumatologique*

**Fig. 5:**
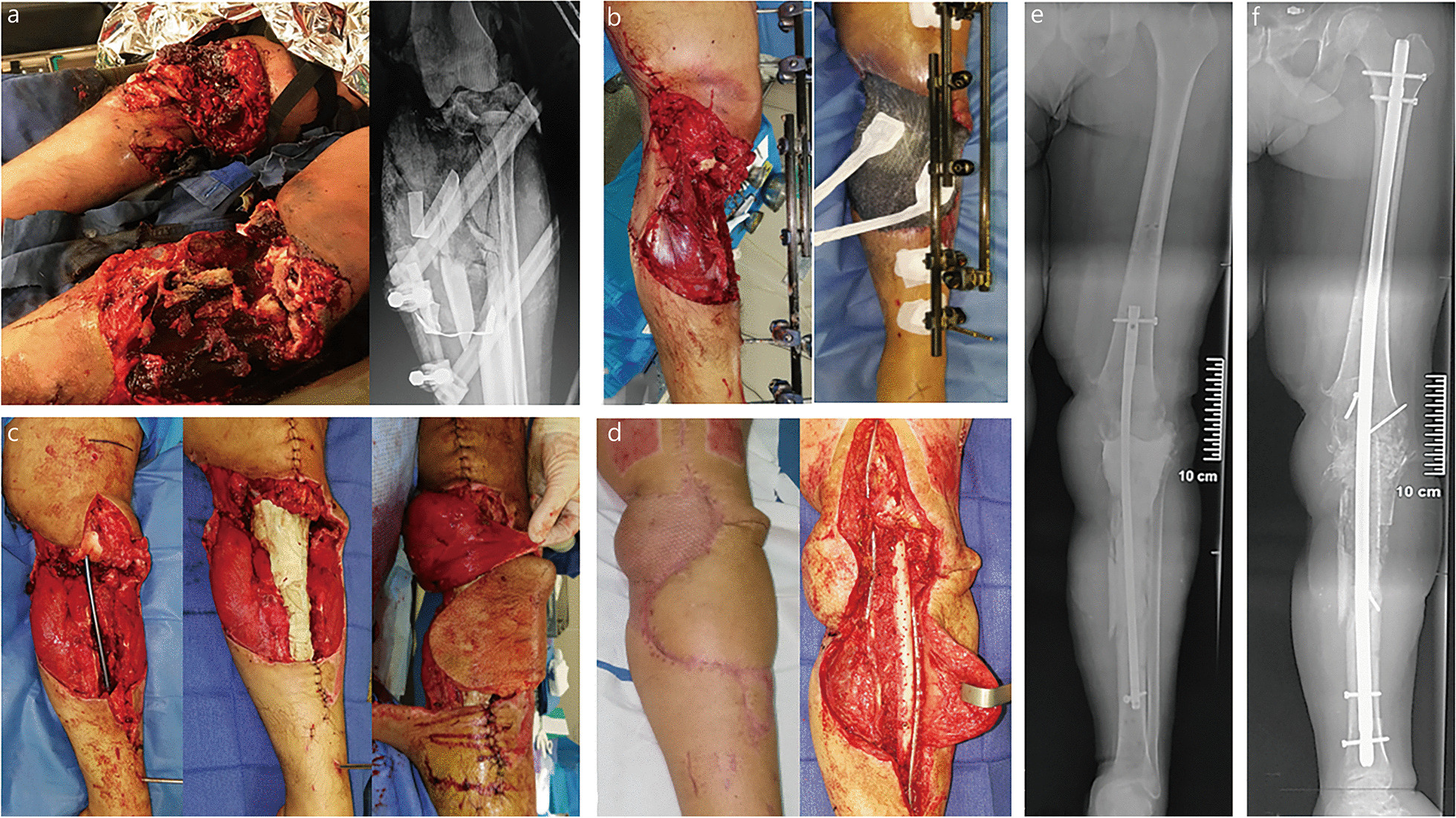
Reconstruction of a massive multi-tissue defect of the left knee and proximal tibia (SOFCOT type 4) following a helicopter crash. **a** Clinical and radiological view at admission. **b** Primary management according to DCO principles. **c** Implantation of a 22 cm-long reinforced spacer and flap coverage with combination of medial gastrocnemius muscle and proximal sural flaps at stage 1 (a free flap transfer was precluded by multiple venous thromboses). **d** Operative views during bone grafting associating cancellous bone grafts and a multiperforated non-vascularized fibular strut. **e** X-rays showing the reinforced spacer after stage 1. **f** Radiological view after stage 2: a knee arthrodesis was performed using a femorotibial nail. Progressive weight bearing was initiated at month-3. Bone union was achieved after 6 months without any complication. DCO damage control orthopedics, SOFCOT *Société Française de Chirurgie Orthopédique et Traumatologique*

**Fig. 6:**
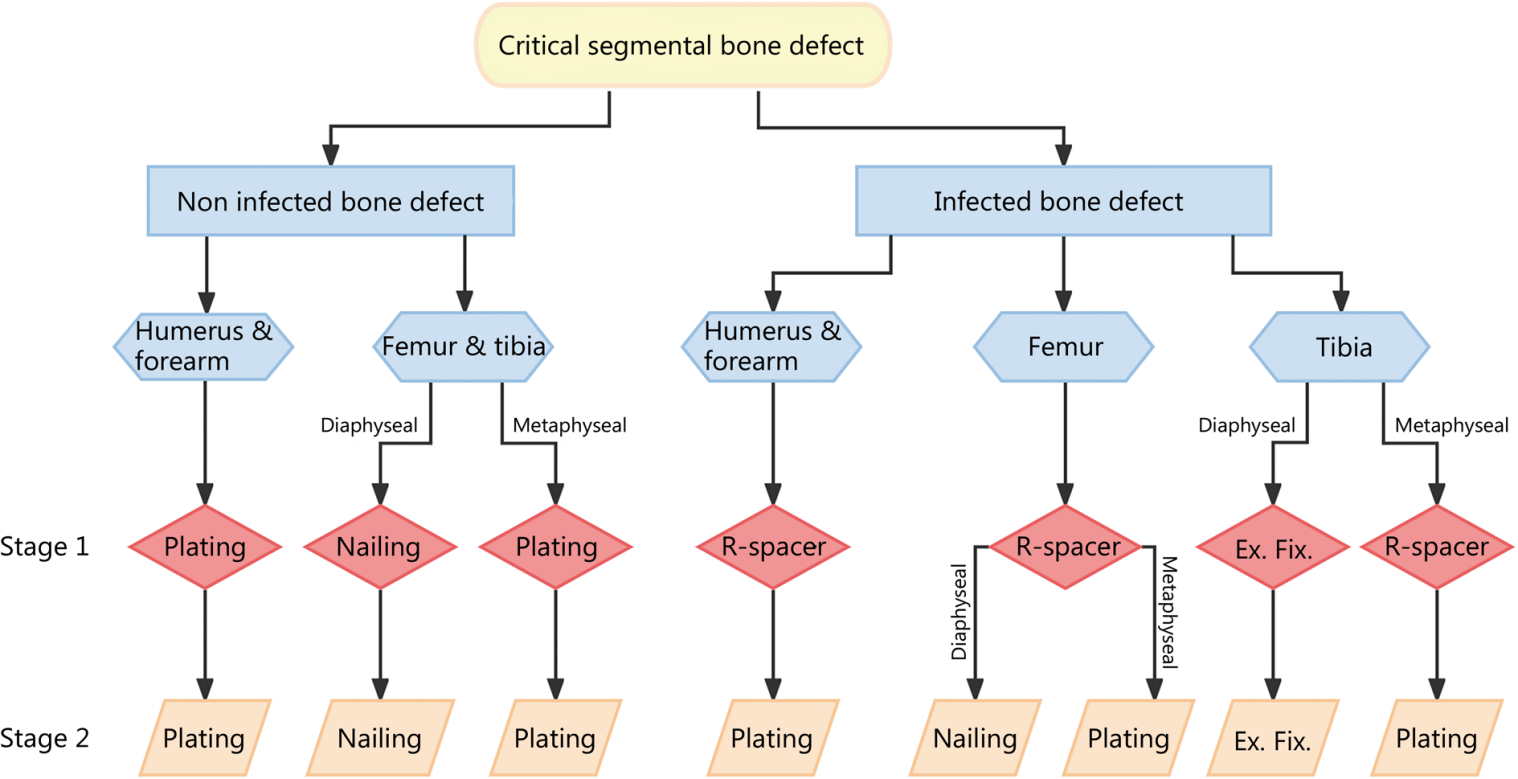
Proposed strategy for bone stabilization in the context of Masquelet technique [31]. Ex. Fix. external fixation, R-spacer reinforced spacer

A lack of mechanical stability in the definitive bone fixation at stage 2 is the second cause of IMT failure [21, 22]. Insufficient stability affects graft revascularization (by rupture of the inner membrane vascular buds), leading to aseptic non-union and subsequent implant failure [9]. If all bone fixations are possible, the implant choice depends on the defect location, associated infection, and surgeon’s preferences. In the absence of infection, intramedullary nailing is mechanically preferred for lower extremity fractures, and plating is preferred for upper extremity fractures and metaphyseal fractures [9]. However, in military practice, most bone defects are primarily stabilized by external fixation and complicated by early infection. Definitive external fixation is naturally compatible with IMT use but exposes to specific issues such as poor patient tolerance, pin-track infection, and insufficient mechanical stability [4, 33]. Therefore, a sequential bone stabilization strategy is often required for a successful IMT application. Conversion from external to internal fixation can be performed in a single procedure during stage 2 despite the risk of pin-track infection between stages [34]. However, a sequential internal fixation, including the use of a reinforced spacer at stage 1, seems preferable for infected bone defect reconstruction in locations where external fixation is not suitable, such as the upper extremity, femur, and tibial metaphysis (Figs. 4, 5 and 6) [4].

### Optimal timing between stages

Basic research of animal found the highest osteo-inductive bioactivity after 2–4 weeks suggesting bone grafting after this short time interval [26, 35]. However, clinical, not evidence-based recommendations usually range from 4 to 8 weeks [7, 9, 34, 35]. In fact, successful outcomes were reported with bone grafting performed several months or years after spacer implantation [36, 37]. Gessmann et al. [35] recently studied the histological properties of IMs from 60 patients at different maturation ages (1–16 weeks). They demonstrated that mesenchymal stem cells and growth factors were found over all time points with minor changes. In addition, according to Masquelet [9], bone grafts at stage 2 can be considered as a foreign body that is likely to reactivate the IM biological properties. Thus, the timing for the second stage should be based on clinical factors rather than on a theoretical optimal stages interval. The only valid rule is to wait for the infection control and the perfect soft tissues healing, particularly in combat-related injuries requiring flap reconstruction [9].

### Reported complications

The IMT global complication rate ranges from 26 to 49% in the literature, with two frequent complications: nonunion and infection [19, 38, 39]. Morelli et al. [38] found 9.6% of additional bone healing procedures after IMT application, and 26.7% of other revision surgeries. Mi et al. [39] compared the IMT results (from their meta-analysis of 41 papers) with those of 37 papers reporting on bone transport. They could not find any significant difference between techniques in terms of bone union, infection, malunion, and amputation; nevertheless, the refracture rate was 8.5 times higher with bone transport. Wen et al. [40] reported a monocentric retrospective study comparing the results of IMT, bone transport, and free vascularized fibular grafting for posttraumatic long-bone defect reconstruction in 317 patients. Overall bone union and complication rates were similar between the three techniques. Thus, IMT achieves similar outcomes to those of other reconstructive procedures in terms of bone union and complication rates but with greater ease of use [21].

## Tibia reconstruction

In the literature, IMT is applied for tibia reconstruction in two-thirds of cases. It is also the site at which the complication rate is the highest, with a clear predominance of infection [38, 39]. Due to the superficial location of this bone, any timing error or complication related to soft-tissue coverage carries the risk of spacer contamination [21]. In a recent meta-analysis, Hsu et al. [41] reported a postoperative infection rate of 24% after tibia reconstruction using IMT. Severe soft-tissue injury, early wound infection, and delayed soft-tissue coverage make secondary tibia nailing risky in military practice [1]. Plating remains preferable for proximal or diaphyseal metaphyseal reconstruction, for which external fixation is poorly tolerated and causes joint stiffness (Fig. 6) [21, 33].

### Diaphyseal tibia defects

Regarding the risk of infection, most diaphyseal tibia defects are stabilized by external fixation in military practice [1, 3, 21]. A modular frame should be preferred to a circular frame to allow soft-tissue reconstruction and delayed bone grafting. A unilateral frame is sufficient during the period of IM induction after stage 1, but a multiplanar frame is required at stage 2 to provide enough stability for bone graft integration (Fig. 2). Conversely, a subsequent progressive frame dynamization is necessary to enhance graft corticalization [3, 9, 21, 33]. Masquelet [8, 9] recommends the performance of tibia reconstructions pressed on the fibula and reinforced by inter-tibiofibular grafts at both ends of the reconstruction. When access to the fibula is easy during stage 1 (e.g., in the case of anterior compartment damage), an inter-tibiofibular spacer should be made (Fig. 2). In other situations where a tibial spacer is used, a bipolar inter-tibiofibular graft is added during stage 2 using an approach selected in accordance with potential vascular injury [21, 33].

Although it runs a higher risk of septic complications, locked intramedullary nailing is the best mechanical option, especially in very large tibia defects (Fig. 5). For Mauffrey et al. [34], nailing could allow early weight-bearing compared to external fixation, which seems questionable within an IMT procedure. Acting as a central core, the intramedullary nail also has the advantage of limiting the bone graft volume inside the membrane. This central part of the graft will be eliminated under the effect of the mechanical constraints during the graft corticalization process, ending with the formation of a new intramedullary canal [33].

### Metaphyseal tibia defects

Proximal and distal tibia reconstructions are best achieved using plating. The primary management of such severe multi-tissue injuries usually requires DCO application with temporary external fixation. In the absence of infection, secondary plating can be performed readily during stage 1 (Fig. 3). Conversely, in cases of infected bone defect, the temporary external fixator should be converted to a reinforced spacer during stage 1 and the definitive internal fixation delayed at stage 2 after achieving extended antibiotic medication [4, 33]. Importantly, distal tibia reconstructions should also be pressed on the fibula to improve mechanical stability (Fig. 4).

## Femur and upper extremity reconstruction

The complications rate is lower at the femur, humerus, and forearm levels, where soft-tissue issues are more uncommon [21]. For mechanical and functional reasons, internal fixation should always be preferred to external fixation, using a reinforced spacer if necessary (Fig. 6) [4, 21, 33].

### Femur defects

Femur mechanical conditions are less favorable for successful graft integration than those of the tibia. The divergence between mechanical and anatomical axes results in high varus loads, jeopardizing graft corticalization in cases of insufficient fixation stability [8, 19]. A stable internal fixation at stage 2 is then critical to achieve bone union (Fig. 1). Morwood et al. [42] reported that patients treated with intramedullary nailing experienced faster union, requiring fewer grafting procedures and fewer reoperations, than those treated by plating. When plate fixation is required at the distal femur, a fibular segment must often be intercalated inside the membrane on the medial aspect of the reconstruction to counteract the bending varus forces. This fibular segment can be a micro-vascularized or non-vascularized free transfer that will be revascularized by the IM [8, 9, 43].

### Upper extremity defects

Humerus, radius, and ulna defects are best stabilized using plates [21, 33, 44]. The involvement of a thick soft-tissue envelope and appropriate stability of plate fixation explain the observation that failures of humerus and forearm reconstructions are infrequent [21, 44]. Humerus reconstruction by IMT may require an anterior transposition of the radial nerve through the fracture or non-union site in stage 1. The surgical approach at stage 2 is then much easier and safer since the transposed nerve is positioned on the medial side of the bone gap and protected by the membrane [45].

The complication rate is higher at the hand, similar to the tibia level. The superficial location of the reconstruction space leads to frequent septic failure secondary to spacer exposure or other soft-tissue coverage issues [21]. Appropriate stable fixation can be achieved by either multiple pinning or plating depending on the defect location (i.e., phalangeal or metacarpal) [12, 46].

## Massive bone defect reconstruction

Combat-related extremity injuries are often characterized by extensive soft-tissue and bone defects caused by high-velocity projectiles or explosive devices. These massive tissue destructions are frequently observed in polytraumatized patients whose associated injuries or early complications do not allow for long and complex reconstructive procedures such as micro-vascularized tissue transfer [2]. Moreover, without exception, these sophisticated procedures are not feasible in the operation theatres [10, 32]. In addition, even under ideal conditions, the management of extensive bone defects remains challenging regarding bone stabilization, available bone stock for grafting, and time to bone union. Therefore, amputation can be considered in these massive multi-tissue defects for which vascularized transfers or bone transport seem inappropriate. In our experience, the Masquelet technique sometimes permits limb salvage in such extreme clinical presentations, provided infection is absent or under control (Fig. 5). These massive reconstructions frequently involve the combination of simultaneous pedicled flaps with a sequential internal fixation.

### Extensive soft-tissue coverage at stage 1

Since micro-vascularized flaps can be hardly achieved on the battlefield or may be contraindicated by associated injuries or vascular complications (e.g., multiple venous thromboses), soft-tissue coverage of extensive defects is performed using simultaneous pedicled flaps [10, 32]. This problem is frequently encountered at the leg level, where local muscle flaps can be combined with local or distant fascio-cutaneous flaps (Fig. 5). In this context, muscle flaps are often preferred for the proximal two- thirds of the tibia (Figs. 2, 3, and 5), but cross-leg flaps are particularly straightforward for distal tibia coverage (Fig. 4). Of course, when feasible, a free latissimus dorsi flap transfer must also be considered.

Considering the large size of the bone defect and the need for dual or triple flap transfers, external fixation is not suitable. Bone stabilization at stage 1 is best achieved using a reinforced spacer with an intramedullary frame (Figs. 4 and 5). A reinforced spacer offers several advantages in this situation: 1) it is a quick and safe procedure that is perfectly suited to complex soft-tissue reconstruction, 2) the risk of bacterial adhesion is low, 3) the easy hardware removal facilitates possible subsequent bone debridement in case of septic recurrence, and 4) there are no risks related to pin-tract infection during the conversion to definitive fixation [4].

### Massive bone grafting with highly stable fixation at stage 2

Reconstruction of large bone defects requires highly stable internal fixation during stage 2. Intramedullary nailing is preferred from a mechanical perspective. This also brings the advantage of limiting the graft volume. However, in cases of extensive bone defect, the mechanical stability obtained with a standard locked nail can be insufficient for bone graft integration within the IMT. In such situations, nailing can be combined with a locking plate or a free fibular strut placed within the membrane [33]. This fibular segment also permits limiting the volume of cancellous bone graft, which can be considerable in cases of metaphyso-diaphyseal defect (Fig. 5). Mixing autograft with allograft or bone substitute is necessary for such massive bone grafting. Conventionally, the ratio of allograft/autograft should not exceed 1:3 because allograft does not contain stem cells or growth factors [9]. However, Pesciallo et al. [47] recently found that a higher proportion of allograft (up to 64%) achieves similar union and failure rates than those reported for similar series that relied on lower allograft proportions. Masquelet [9] also stressed that the membrane cavity must be fully filled to avoid down-migration of the graft due to gravity in a standing position and subsequent proximal non-union.

The “Capasquelet” is an alternative strategy described by Combal et al. [48] for reconstruction of femoral bone defects exceeding 10 cm. This two-stage hybrid approach combines the Masquelet technique and the allograft technique with inlay of a vascularized fibula (i.e., Capanna technique) [48, 49]. At stage 2, a vascularized fibular autograft is embedded in a prepared fresh femoral allograft with a proximal and distal overlap of 2 cm. Stable bone stabilization is obtained by the combination of this inlay bone graft with a locking plate. The preliminary results of this innovative technique are encouraging with bone union being achieved within a short time, thereby allowing early full weight bearing [48]. However, as it requires femoral allograft access and microsurgery skills, the “Capasquelet” might not be the easiest solution in a combat-related situation due to limited resources, with associated soft tissue and potential vascular lesions.

## Bone reconstruction in the field

Contrary to micro-vascularized bone transfers or bone transport procedures, the Masquelet technique can be applied with limited resources in the austere environment of forward surgical units or improvised field hospitals [12, 15, 17, 22]. Although several barriers may impede its application, we believe that IMT is the best option for large bone defect reconstruction in such settings. Various technical tricks help to avoid these obstacles and achieve bone union.

### Challenge 1: infection control

This is the main difficulty on the battlefield due to delayed surgical management with limited resources. Stage 0 must include serial debridement sessions with appropriate antibiotic medication and negative wound pressure therapy to avoid iterative environmental contamination [4, 43]. Next, a stable soft-tissue envelope must be reconstructed at stage 1, considering that the choices for soft-tissue coverage methods are always restricted in this setting [10]. In addition to these surgical challenges, it is also crucial to have laboratory resources permitting bacteriological cultures with precise germ identification and to be capable of providing an adapted and extended antibiotic treatment. In the Sahel, we found that the latter point was a frequent issue when managing local patients with combat-related bone defects [22].

### Challenge 2: limitation in surgical cement

PMMA cement can be lacking in forward surgical units that are not dedicated to performing bone reconstruction. Similarly, the absence of PMMA often precludes the use of the Masquelet technique in developing countries [20]. To avoid this obstacle, Mozumder et al. [50] achieved IMT using a polypropylene spacer made from disposable syringes in Bangladesh. They observed results comparable with those obtained with a PMMA spacer for long-bone reconstruction [50]. Successful metacarpal bone reconstructions were also achieved using disposable syringes as spacers in a forward surgical unit [12]. A recent experimental study demonstrated that polypropylene spacers induce membrane encapsulation with histologic characteristics and bone regenerative capacities that seem like those of PMMA spacers. These findings support the possibility of a cementless Masquelet technique in cases of PMMA shortage caused by a lack of resources [20].

### Challenge 3: limitation in available bone stock

Allograft, bone substitute, or sophisticated bone harvesting devices, such as the reamer/irrigator/aspirator (RIA), are usually not available in battlefield medical treatment facilities [43, 51]. As a result, management of large long-bone defects can be compromised by the lack of autograft during stage 2. However, various technical tricks permit limiting the required volume of cancellous graft.

The use of intramedullary nailing is critically important when the patient’s bone stock is limited or the defect is extensive. The nail acts like a central core limiting the bone graft volume to be applied inside the membrane. When intramedullary nailing is not suitable, a central fibular strut is an interesting option limiting the cancellous bone graft quantity and adding mechanical stability to the bone fixation method, be it for external fixation or plating (Figs. 3 and 5).

A non-vascularized fibular graft combined with IMT seems to be a valid alternative to the conventional micro-vascularized fibular bone transfer [8, 52–55]. The membrane ensures progressive graft revascularization, avoiding the pitfalls of non-vascularized fibular graft. Using this technique, Fitoussi and Ilharreborde [53] obtained satisfying results for the reconstruction of large bone defects in children. Masquelet [8] previously reported similar outcomes in adult patients but faced late stress fracture, which suggested insufficient graft revascularization. He subsequently improved the technique by performing multiple perforations in the fibular graft, using a 2-mm drill to permit penetration of the vascular buds generated by the membrane. Several African authors have recently reported similar results confirming the relevance of this technique in low-resource settings [54, 55]. Thus, the combination of a multiperforated fibular graft with cancellous autografts in an IM seems to be a reliable strategy to achieve bone union in local patients treated in the field for large bone defects (Figs. 3 and 5).

Under certain conditions, limb shortening may be considered to limit the extent of the bone defect. At the humerus, a 3–4 cm shortening can be tolerated without significant functional impairment (Table 1) [6]. In the lower extremity, femur or tibia shortening must remain the exception and should not exceed 2 cm. However, such shortening should probably be contraindicated at the tibia level when the fibula is intact, since the fibula should be used as a support for tibia reconstruction [3, 6, 8, 9, 43].

## Future directions to improve Masquelet technique outcomes

Various approaches are currently being developed worldwide to refine IMT and promote its efficiency. These approaches mainly focus on four different aspects: 1) substitution of the standard PMMA spacer with another biomaterial able to generate an IM exhibiting at least similar osteogenic properties to those conferred by PMMA, 2) enhancement of the quality and the viability of ABG implanted into the IM cavity, 3) substitution or combination of ABG with synthetic scaffolds presenting increased osteogenic properties, and 4) IMT conversion into a single-step procedure by performing an “off the shell” membrane strategy.

### Alternative membrane inductors

Various biomaterials have been evaluated for their capacity to generate osteogenic IM in animal models as alternatives to PMMA: smooth or roughened titanium [56], silicone [57], calcium sulfate spacers [58], and polypropylene syringe bodies [20]. Interestingly, Toth et al. [56] determined whether spacer material modification or topography alteration could change biochemical IM environment or bone reconstruction efficiency. By inducing IM in rat models using smooth or roughened PMMA and titanium, they showed that titanium induced membranes were around 35% thicker than PMMA (with better barrier properties), and the inflammatory factor IL-6 was around 35% higher in the roughened groups than smooth groups, potentially suggesting indirect repair enhancement. They concluded that smooth PMMA-IMs promoted better bone regeneration than the three other groups. According to Sagardoy et al. [57], silicone spacers inserted in rat bone defects generated IM with similar biological and histological properties (IM thickness, vessel density, BMP2 and VEGF content) to PMMA-triggered IM. Furthermore, the authors noted that silicone spacers were easier to remove than PMMA spacers at the end of IM induction time. In an 8-week study in an IMT rat model, Ma et al. [58] demonstrated that morphological characteristics of IM around calcium sulfate spacers were similar to those around PMMA, whatever the considered kinetic time. However, they suspected that calcium sulfate-IMs were thicker than PMMA-IMs and that even if not significant, endochondral ossification and higher expression levels of VEGF, TGF-1, and BMP-2 were detected in calcium sulfate-IM compared to PMMA-IM. Lastly, it was recently found that polypropylene-IM exhibited similar histologic characteristics and bone regenerative capacities to PMMA–IM [12, 20]. Overall, from the military surgery point of view and to answer recurrent clinical needs of orthopedic surgeons, a spacer that is easy to handle or insert into the bone defect (in stage 1) and convenient to extract from the IM cavity (in stage 2) will be preferred in the future.

### Autologous bone grafting optimization

Sparse clinical investigations have compared bone union rates obtained with IMT using ABG collected from iliac crests and the RIA technique. Globally, a similar union rate was observed in both groups, but the graft volume collected from RIA was slightly higher (average 47 cm^3^ vs. 37 cm^3^, respectively), with few postoperative complications (including iatrogenic femur fracture) [7, 38, 51]. In an IMT rat model, Sun et al. [59] modified the graft harvest technique to reduce the cortical content of the graft and minimize the graft preparation time. They found that these modifications achieved a bone union rate much higher than that obtained using conventional graft harvesting (92% vs. 36%). Thus, scrupulous graft preparation could also ameliorate the outcome of the IMT procedure in patients.

The addition of biological additives (cells or growth factors) to ABG during stage 2 was proposed to support new bone formation [60]. Experimental studies demonstrated improved bone union after incorporation of concentrated growth factors or BMP-7 in rabbits [61, 62]. However, in clinical practice, the effects of supplementing grafts with BMP-2 or BMP-7 are unclear and growth factors utilization is entirely dependent on surgeon preference [60]. The addition of recombinant growth factors is in fact debated since no clinical comparative study has proven their value so far. Masquelet and Bégué [63] have observed that it could be deleterious to associate BMPs with the graft material inside the IM. Localized high density of the product and possible effects of competition with growth factors secreted by the membrane can lead to partial resorption of the graft [9].

Lastly, Luangphakdy et al. [64] found in a caprine model that a simple scrapping of the IM after spacer removal and prior to grafting may improve healing of segmental bone defects. This removal of the IM inner layer induced bleeding while preserving the mechanical and biological function of the rest of the membrane. Micro-CT showed that scrapping almost double the amount of total bone within the defect 12 weeks post-grafting.

### Synthetic scaffolds

One of the most exciting new areas is the convergence of the Masquelet technique with scaffold guided bone engineering. Scaffolds provide attractive alternatives to conventional bone grafting serving as three-dimensional structures to guide cell migration, proliferation, and differentiation. In load bearing tissues, they also serve as temporary mechanical support structures [65]. The ability of 3D-printing allows the design and manufacture of osteoconductive scaffolds which are optimized for clinical translation in terms of pore size, layering, and degradation [66, 67]. Mainly to overcome graft limitation quantity, a huge diversity of synthetic scaffolds has been developed in preclinic models to be mixed with the ABG during stage 2 [62]. Some of them have been tested clinically [68–71]. Tetsworth et al. [68] achieved very acceptable outcomes in 5 cases of femoral bone defects treated by patient-specific 3D printed titanium cages. Van Vugt et al. [69] implanted a combination of bioactive glass and bone marrow aspirate concentrate in 4 patients who all exhibited complete bone repair and functional recovery. In a larger series, Gupta et al. [70] treated 42 patients with postinfective segmental bone defects using ABG mixed with beta tri-calcium phosphate (B-TCP)-based composite ceramic (34 patients) or ABG alone (15 patients). They found that B-TCP is an efficacious bone-graft expander in the IMT despite the bone union rate of this group was lower (81% vs. 100% with index bone grafting). Always to reduce the required amount of bone graft, but also to main graft position and shape, Cho et al. [71] used a circumferential graft around an absorbable gelatin sponge (the gelatin sponge core was about 21.4% of the bone defect volume) leading to an 86% bone healing rate in 21 patients.

### Toward a single-stage Masquelet technique

To allow a quicker healing time with fewer surgical procedures for the patient, a strategy could be to bypass the membrane induction time by applying a commercial off-the-shelf membrane concomitantly to the bone graft. Interestingly, maxillofacial surgery already uses such a strategy, better known as guided bone regeneration (GBR). In GBR, the implantation of membranes made of natural (collagen, chitosan, alginate) or synthetic polymers [resorbable aliphatic polyesters, including polylactic acid and polycaprolactone or non-resorbable polytetrafluorethylene (PTFE)] in combination with grafting materials is commonly applied to treat peri-implant bone deficiencies or alveolar bone reconstruction. GBR membranes act as passive membranes hindering the soft-tissue invasiveness of the defect. Emerging data also suggest that GBR membranes may bioactively contribute to bone regeneration [72]. The application of GBR membranes to replace the Masquelet membrane for the treatment of long-bone defects has gained some attention in the last ten years. For example, in a rabbit model, Tarchala et al. [73] compared the healing efficiency of ulna bone defects treated by a standard IM or a synthetic non-resorbable PTFE membrane filled with allograft. The bone defect repair was similar in both experimental arms, underlying the promising potential for the treatment of synthetic PTFE membranes. However, because PTFE is a non-absorbable material, more experimental studies using absorbable membranes are needed to validate the streamlining toward a single-stage IMT procedure. In this context, a French research team examined the use of human amniotic membranes as Masquelet membrane alternatives. Human amniotic membranes and Masquelet IM share similar biological properties [74–76]: both are highly organized tissues containing growth factors such as VEGF and TGF-β1, expressing anti-inflammatory proteins and displaying osteogenic properties. Recently, Fenelon et al. [77] showed that no difference existed in the bone regeneration potential of femoral critical-size defects in rats between the two-step IM procedure and the single-step approach using human amniotic membranes.

All these future directions are promising approaches to improving IMT outcomes. However, in a military practice context, surgeons often deal with limited resources. Thus, valuable replacement solutions to the IMT standard of care could include ready-to-use implantable medical devices presenting large-scale manufacturing facilities, easy sterilization processes, and convenient storage modes. We believe disposable polypropylene syringe bodies are an example of a promising alternative spacer to standard PMMA cements in IMT in a military practice context or low-resource environment.

## Conclusions

The Masquelet IMT offers a successful possibility to solve long-bone defects encountered in military practice. Reconstruction of combat-related bone defects often requires prior infection control and soft-tissue reconstruction during stage 1. Providing those requirements are met, IMT even allows bone reconstruction in the austere environment of field medical treatment facilities. However, specific surgical tactics, such as combined inter-tibiofibular grafting or sequential internal fixation, may be required. We believe that this simple, reliable, and replicable procedure should be considered as a technique of choice for the reconstruction of multi-tissue defects resulting from missile or blast injuries.

## Data Availability

Not applicable.

## Abbreviations

ABG: Autologous bone graft
BMP-2: Bone morphogenic protein-2
BMP-7: Bone morphogenic protein-7
B-TCP: Beta tri-calcium phosphate
DCO: Damage control orthopedics
GBR: Guided bone regeneration
IM: Induced membrane
IMT: Induced membrane technique
PMMA: Polymethylmethacrylate
PTFE: Polytetrafluorethylene
RIA: Reamer/irrigator/aspirator
TGF-β1: Transforming growth factor-β1
VEGF: Vascular endothelial growth factor

## References

[1] Murray CK, Hsu JR, Solomkin JS, Keeling JJ, Andersen RC, Ficke JR (2008). Prevention and management of infections associated with combat related extremity injuries. J Trauma.

[2] Mathieu L, Bazile F, Barthélémy R, Duhamel P, Rigal S (2011). Damage control orthopaedics in the context of battlefield injuries: the use of temporary external fixation on combat trauma soldiers. Orthop Traumatol Surg Res.

[3] Mathieu L, Bilichtin E, Durand M, de l’Escalopier N, Murison JC, Collombet JM (2020). Masquelet technique for open tibia fractures in a military setting. Eur J Trauma Emerg Surg.

[4] Mathieu L, Tossou-Odjo L, de l’Escalopier N, Demoures T, Baus A, Brachet M (2020). Induced membrane technique with sequential internal fixation: use of a reinforced spacer for reconstruction of infected bone defects. Int Orthop.

[5] Baus A, Bich CS, Grosset A, de Rousiers A, Duhoux A, Brachet M (2020). Medical and surgical management of lower extremity war-related injuries. Experience of the French military health service (FMHS). Ann Chir Plast Esthet.

[6] Masquelet AC, Sales de Gauzy J, Bauer T, Fabre A, Fitoussi F, Hannouche D (2012). Reconstruction des pertes de substance osseuse diaphysaires d’origine traumatique. Stratégies, recommandations, perspectives. Rev Chir Orthop Traumatol.

[7] Giannoudis PV, Harwood PJ, Tosounidis T, Kanakaris NK (2016). Restoration of long bone defects treated with the induced membrane technique: protocol and outcomes. Injury.

[8] Masquelet AC, Fitoussi F, Bégué T, Muller GP (2000). Reconstruction of the long bones by the induced membrane and spongy autograft. Ann Chir Plast Esthet.

[9] Masquelet AC (2017). Induced membrane technique: pearls and pitfalls. J Orthop Trauma.

[10] Mathieu L, Plang S, de l’Escalopier N, Murison JC, Gaillard C, Bertani A (2021). Extremity soft tissue coverage in the combat zone: use of pedicled flap transfers by the deployed orthopedic surgeon. Mil Med Res.

[11] Rigal S, de l’Escalopier N, Mathieu L (2018). Temporary fixation of limbs and pelvis. Orthop Traumatol Surg Res.

[12] Murison JC, Pfister G, Amar S, Rigal S, Mathieu L (2019). Metacarpal bone reconstruction by a cementless induced membrane technique. Hand Surg Rehabil.

[13] Franke A, Hentsch S, Bieler D, Schilling T, Weber W, Johann M (2017). Management of soft-tissue and bone defects in a local population: plastic and reconstructive surgery in a deployed military setting. Mil Med.

[14] Knipper P, Bégué T, Pasquesoone L, Guerre E, Khonsari R, Girard P (2021). Plastic surgery and fighting: our experience during Nagorno-Karabakh war in 2020. Ann Chir Plast Esthet.

[15] Mathieu L, Masquelet AC (2019). Use of the induced membrane technique for long bone reconstruction in low-resource settings. Med Sante Trop.

[16] Durand M, Barbier L, Mathieu L, Poyot T, Demoures T, Souraud JB (2020). Towards understanding therapeutic failures in Masquelet surgery: first evidence that defective induced membrane properties are associated with clinical failures. J Clin Med.

[17] Bilichtin E, de Rousiers A, Durand M, de l’Escalopier N, Collombet JM, Rigal S (2020). Bone reconstruction by the induced membrane technique. What differences between conventional and ballistic trauma?. Orthop Traumatol Surg Res.

[18] Mathieu L, Potier L, Ndiaye R, Choufani C, Mbaye E, Niang CD (2020). Challenges of the induced-membrane technique in the reconstruction of traumatic tibial defect with limited resources: a cohort study. Acta Orthop Belg.

[19] Mathieu L, Durand M, Demoures T, Steenman C, Masquelet AC, Collombet JM (2020). Repeated induced-membrane technique failure without infection: a series of three consecutive procedures performed for a single femur defect. Case Rep Orthop.

[20] Mathieu L, Murison JC, de Rousiers A, de l’Escalopier N, Lutomski D, Collombet JM (2021). The Masquelet technique: can disposable polypropylene syringes be an alternative to standard PMMA spacers? A rat bone defect model. Clin Orthop Relat Res.

[21] Mathieu L, Durand M, Collombet JM, de Rousiers A, de l'Escalopier N, Masquelet AC (2021). Induced membrane technique: a critical literature analysis and proposal for a failure classification scheme. Eur J Trauma Emerg Surg.

[22] Choufani C, Demoures T, de l'Escalopier N, Chapon MP, Barbier O, Mathieu L (2022). Application of the Masquelet technique in austere environments: experience from a French forward surgical unit deployed in Chad. Eur J Trauma Emerg Surg.

[23] Anderson JM, Rodriguez A, Chang DT (2008). Foreign body reaction to biomaterials. Semin Immunol.

[24] Viateau V, Guillemin G, Calando Y, Logeart D, Oudina K, Sedel L (2006). Induction of a barrier membrane to facilitate reconstruction of massive segmental diaphyseal bone defects: an ovine model. Vet Surg.

[25] Gouron R, Petit L, Boudot C, Six I, Brazier M, Kamel S (2017). Osteoclasts and their precursors are present in the induced-membrane during bone reconstruction using the Masquelet technique. J Tissue Eng Regen Med.

[26] Pelissier Ph, Masquelet AC, Bareille R, Mathoulin Pelissier S, Amedee J (2004). Induced membranes secrete growth factors including vascular and osteoinductive factors and could stimulate bone regeneration. J Orthop Res.

[27] Wang W, Zuo R, Long H, Wang Y, Zhang Y, Sun C (2020). Advances in the Masquelet technique: myeloid-derived suppressor cells promote angiogenesis in PMMA-induced membranes. Acta Biomater.

[28] Tang Q, Tong M, Zheng G, Shen L, Shang P, Liu H (2018). Masquelet’s induced membrane promotes the osteogenic differentiation of bone marrow mesenchymal stem cells by activating the Smad and MAPK pathways. Am J Transl Res.

[29] Shah SR, Smith BT, Tatara AM, Molina ER, Lee EJ, Piepergerdes TC (2017). Effets of local antibiotic delivery from porous space maintainers on infection clearance and induction of an osteogenic membrane in an infected bone defect. Tissue Eng Part A.

[30] Sanchez CJ, Ward CL, Romano DR, Hurtgen BJ, Hardy SK, Woodbury RL (2013). *Staphylococcus aureus* biofilms decrease osteoblast viability, inhibits osteogenic differentiation, and increases bone resorption in vitro. BMC Musculoskelet Disord.

[31] Zhang C, Zhu C, Yu G, Deng K, Yu L (2020). Management of infected bone defects of the lower extremities by three-stage induced membrane technique. Med Sci Monit.

[32] Mathieu L, Gaillard C, Pellet N, Bertani A, Rigal S, Rongiéras F (2014). Soft tissue coverage of war extremity injuries: the use of pedicle flap transfers in a combat support hospital. Int Orthop.

[33] Mathieu L, Masquelet AC (2020). La stabilisation instrumentale: avantages et implications. La technique de la membrane induite: principes, pratique et perspectives.

[34] Mauffrey C, Hake ME, Chadayammuri V, Masquelet AC (2016). Reconstruction of long bone infections using the induced membrane technique: tips and tricks. J Orthop Trauma.

[35] Gessmann J, Rosteius T, Baecker H, Sivalingam K, Peter E, Schildhauer TA (2021). Is the bioactivity of induced membranes time dependent?. Eur J Trauma Emerg Surg.

[36] Gindraux F, Loisel F, Bourgeois M, Oudina K, de Billy B, Sergent P (2020). Induced membrane maintains its osteogenic properties even when the second stage of Masquelet’s technique is performed later. Eur J Trauma Emerg Surg.

[37] Tacharla M, Harvey EJ, Barralet J (2016). Biomaterial-stabilized soft tissue healing for healing of critical-sized bone defects: the Masquelet technique. Adv Healthc Mater.

[38] Morelli I, Drago L, George DA, Gallazzi E, Scarponi S, Romanò CL (2016). Masquelet technique: myth or reality? A systematic review and meta-analysis. Injury.

[39] Mi M, Papakostidis C, Wu X, Giannoudis PV (2020). Mixed results with the Masquelet technique: A fact or a myth?. Injury.

[40] Wen G, Zhou R, Wang Y, Lu S, Chai Y, Yang H (2019). Management of post-traumatic long bone defects: a comparative study based on long-term results. Injury.

[41] Hsu CA, Chen SH, Chan SY, Yu YH (2020). The induced membrane technique for the management of segmental tibial defect or nonunion: a systematic review and meta-analysis. Biomed Res Inter..

[42] Morwood MP, Streufert BD, Bauer A, Olinger C, Tobey D, Beebe M (2019). Intramedullary nails yield superior results compared with plate fixation when using the Masquelet technique in the Femur and Tibia. J Orthop Trauma.

[43] Mathieu L, Masquelet AC (2020). Utilisation de la technique en conditions précaires: trucs et astuces. La technique de la membrane induite: principes, pratique et perspectives.

[44] Braswell MJ, Bulloch LR, Gaston RG, Garcia RM (2022). Outcomes after use of the induced membrane technique for fractures of the upper extremity. J Hand Surg Am.

[45] Gaillard J, Masquelet AC, Boutroux P, Cambon-Binder A (2020). Induced-membrane treatment of refractory humeral non-union with or without bone defect. Orthop Traumatol Surg Res.

[46] Moris V, Loisel F, Cheval D, See LA, Tchurukdichian A, Pluvy I (2016). Functional and radiographic evaluation of the treatment of traumatic bone loss of the hand using the Masquelet technique. Hand Surg Rehab.

[47] Pesciallo CA, Garabano G, Dainotto T, Ernst G (2021). Masquelet technique in post-traumatic infected femoral and tibial segmental bone defects. Union and reoperation rates with high proportions (up to 64%) of allograft in the second stage. Injury.

[48] Combal A, Thuau F, Fouasson-Chailloux A, Arrigoni PP, Baud’huin M, Duteille F (2021). Preliminary results of the “Capasquelet” technique for managing femoral bone defects—combining a Masquelet induced membrane and Capanna vascularized fibula with an allograft. J Pers Med.

[49] Capanna R, Campanacci DA, Belot N, Beltrami G, Manfrini M, Innocenti M (2007). A new reconstructive technique for intercalary defects of long bones: the association of massive allograft with vascularized fibular autograft long-term results and comparison with alternative techniques. Orthop Clin N Am.

[50] Mozumder M, Chowdhury A, Islam M. Induced membrane technique (Masquelet technique) for the treatment of bone defect using piece of disposable syringe as a spacer in developing countries. 37th SICOT Orthopaedic World Congress, 2016, abstract no: 43308. Available at: http://www.sicot.org/sites/default/files/images/Rome/Abstract-Book-Free-Papers.pdf. Accessed 1 July 2020.

[51] Stafford PR, Norris BL (2010). Reamer-irrigator-aspirator bone graft and bi Masquelet technique for segmental bone defect nonuions: a review of 25 cases. Injury.

[52] Beris AE, Lykissas MG, Korompilias AV, Vekris MD, Mitsionis GI, Malizos KN (2011). Vascularized fibula transfer for lower limb reconstruction. Microsurgery.

[53] Fitoussi F, Ilharreborde B (2015). Is the induced-membrane technique successful for limb reconstruction after resecting large bone tumors in children?. Clin Orthop Relat Res.

[54] Bhosale AH (2021). Reconstruction of a post-traumatic ibia defect of 10 cm in a 6-month-old induced membrane by non-vascularized fibula autograft—A case report. Trauma Case Rep.

[55] Yaokreh JB, Yapo Kouamé GS, Odéhouri-Koudou TH, Ouattara O (2022). Induced membrane technique for reconstruction of a 25 humerus diaphyseal defect secondary to chronic osteomyelitis in an adolescent. Afr J Paediatr Surg.

[56] Toth Z, Roi M, Evans E, Watson JT, Nicolaou D, McBride-Gagyi S (2019). Masquelet technique: effects ofspacer material and micro-topography on factor expression and bone regeneration. Ann Biomed Eng.

[57] Sagardoy T, Ehret C, Bareille R, Benoit J, Amédée J, De Mones E (2018). Influence of external beam radiotherapy on the properties of PMMA versus silicone induced membranes in a bilateral segmental bone defect in rats. Tissue Eng Part A.

[58] Ma YF, Jiang N, Zhang X, Qin CH, Wang L, Hu YJ (2018). Calcium sulfate induced versus PMMA-induced membrane in a critical-sized femoral defect in a rat model. Sci Rep.

[59] Sun H, Godbout C, Ryan G, Hoit G, Higgins J, Schemitsch EH (2022). The induced membrane technique: optimization of bone grafting in a rat model of segmental bone defect. Injury.

[60] Alford AI, Nicolaou D, Hake M, McBride-Gagyi S (2021). Masquelet’s induced membrane technique: review of current concepts and future directions. J Orthop Res.

[61] Karimi Ghahfarrokhi E, Meimandi-Parizi A, Oryan A, Ahmadi N (2021). Effects of combination of BMP7, PFG, and autograft on healing of the experimental critical radial bone defect by induced membrane (Masquelet) technique in rabbit. Arch Bone Jt Surg.

[62] Arıcan G, Özmeriç A, Fırat A, Kaymaz F, Ocak M, Çelik HH (2022). Micro-ct findings of concentrated growth factors (CGF) on bone healing in Masquelet technique - an experimental study in rabbits. Arch Orthop Trauma Surg.

[63] Masquelet AC, Bégué T (2010). The concept of induced membrane technique for reconstruction of long bone defects. Orthop Clin N Am.

[64] Luangphakdy MS, Pluhar E, Piuzzi NS, D'Alleyrand JC, Carlson CS, Bechtold JE (2017). The effect of surgical techique and spacer texture on none regeneration: a caprine study using the Masquelet technique. Clin Orthop Relat Res.

[65] Henkel J, Woodruff MA, Epari DR, Steck R, Glatt V, Dickinson IC (2013). Bone regeneration based on tissue engineering conceptions—a 21st century perspective. Bone Res.

[66] Chen H, Han Q, Wang C, Liu Y, Chen B, Wang J (2020). Porous scaffold design for additive manufacturing in orthopedics: a review. Front Bioeng Biotechnol.

[67] Kobbe P, Laubach M, Hutmacher DW, Alabdulrahman H, Sellei RM, Hildebrand F (2020). Convergence of scaffold-guided bone regeneration and RIA bone grafting for the treatment of a critical-sized bone defect of the femoral shaft. Eur J Med Res.

[68] Tetsworth K, Woloszyk A, Glatt V (2019). 3D printed titanium cages combined with the Masquelet technique for the reconstruction of segmental femoral defects: preliminary clinical results and molecular analysis of the biological activity of human-induced membranes. OTA Int.

[69] Van Vugt TAG, Geurts JAP, Blokhuis TJ (2021). Treatment of infected tibial non-unions using a BMAC and S53P4 BAG combination for reconstruction of segmental bone defects: a clinical case series. Injury.

[70] Gupta S, Malhotra A, Jindal R, Garg SK, Kansay R, Mittal N (2019). Role of beta tri-calcium phosphate based composite ceramic as bone graft expander in Masquelet’s induced membrane technique. Indian J Orthop.

[71] Cho JW, Kim J, Cho WT, Kim JK, Song JH, Kim HJ (2017). Circumferential bone grafting around an absorbable gelatin sponge core reduced the amount of grafted bone in the induced membrane technique for critical-size defects of long bones. Injury.

[72] Omar O, Elgali I, Dahlin C, Thomsen P (2019). Barrier membranes: More than the barrier effect?. J Clin Periodontol.

[73] Tarchala M, Engel V, Barralet J, Harvey EJ (2018). A pilot study: alternative biomaterials in critical sized bone defect treatment. Injury.

[74] Gindraux F, Rondot T, de Billy B, Zwetyenga N, Fricain JC, Pagnon A (2017). Similarities between induced membrane and amniotic membrane: novelty for bone repair. Placenta.

[75] Grzywocz Z, Pius-Sadowska E, Klos P, Gryzik M, Wasilewska D, Aleksandrowicz B (2014). Growth factors and their receptors derived from human amniotic cells in vitro. Folia Histochem Cytobiol.

[76] Litwiniuk M, Radowicka M, Krejner A, Śladowska A, Grzela T (2018). Amount and distribution of selected biologically active factors in amniotic membrane depends on the part of amnion and mode of childbirth. Can we predict properties of amnion dressing? A proof-of-concept study. Cent Eur J Immunol.

[77] Fenelon M, Etchebarne M, Siadous R, Grémare A, Durand M, Sentilhes L (2021). Comparison of amniotic membrane versus the induced membrane for bone regeneration in long bone segmental defects using calcium phosphate cement loaded with BMP-2. Mater Sci Eng C Mater Biol Appl.

